# The effectiveness of varicocele embolisation for the treatment of varicocele related orchalgia

**DOI:** 10.1186/s40064-015-1177-2

**Published:** 2015-08-04

**Authors:** David W Muthuveloe, Vinnie During, Daniel Ashdown, Nicholas J Rukin, Rob G Jones, Prashant Patel

**Affiliations:** 1 School of cancer sciences, University of Birmingham, Birmingham, UK; 2 Cambridge Health at Work, Cambridge Biomedical Campus, Hills Road, Cambridge, UK; 3 The Royal Wolverhampton Hospital NHS Trust, Wolverhampton, UK; 4 Interventional Radiology Department, University Hospitals Birmingham, Birmingham, UK

**Keywords:** Varicocele, Varicocele embolization, Varicocele related orchalgia

## Abstract

**Purpose:**

Orchalgia is a common problem with varicoceles however the association between varicocele embolisation for the treatment of varicocele related pain has not been widely investigated. We aim to investigate the effectiveness of varicocele embolisation for the treatment of orchalgia secondary to varicoceles; and to see if pre-embolisation pain scores can be used to predict treatment outcomes.

**Methods:**

A prospectively collected database of patients undergoing varicocele embolisation for pain was analysed over a 10-year period. Pain scores were assessed with a 10-point visual analogue score. Analgesia requirements and satisfaction scores were assessed with questionnaires.

**Results:**

Total of 96 cases. Median age was 34 years old. Median pain scores reduced significantly following embolisation (p < 0.001). 74% had reduced pain (30% of these had resolution of pain), 24% had no change in symptoms and 1% had worsening pain. Those with moderate or severe pain had a reduction of pain in 81 and 79% of cases respectively, however 64% of cases with mild pain did not experience any benefit. We also noted a reduction in analgesia requirements and a median satisfaction score of 8/10.

**Conclusion:**

Primary varicocele embolisation can successfully reduce varicocele related orchalgia. It works best in those with moderate or severe pain. The majority of patients with mild pain may not experience any benefit so should be counseled appropriately. The classification of patients into those with mild, moderate or severe symptoms prior to embolisation should be done, so robust consenting can be performed.

## Background

Varicoceles are abnormal dilated and incompetent veins within the pampiniform plexus of the spermatic cord (McAninch and Lue 2012). The incidence of varicoceles is 15% in the normal male population and 40% in patients with male factor infertility, with 75–95% being left sided (Practice Committee of the American Society for Reproductive Medicine 2004; Köse et al. 2013). Orchalgia is associated with varicoceles, with the pain being classically described as a dull, throbbing pain, which is exacerbated by straining and standing for long periods. Up to 10% of men with varicoceles complain of scrotal pain and in 2–14% of men with chronic scrotal pain the cause is varicoceles (Kass and Marcol 1992; Peterson et al. 1998).

Embolisation techniques have been successfully used to treat varicoceles, often employing platinum based coils. Currently most embolisation coils are MRI compatible. Fibers on the coil help stimulate thrombosis of the vein, which leads to vein occlusion (Iaccarino and Venetucci 2012). With radiological guidance, coils can be placed accurately and safely, enabling accurate placement to prevent recurrence (Nabi et al. 2004). The recurrence rate from embolisation is approximately 5%, with a complication rate of 10% (Bechara et al. 2009). This is comparable to open varicocelectomy, which had an overall recurrence rate of up to 17% and a complication rate of 30% (Al-Said et al. 2008), and laparoscopic varicocelectomy that has a recurrence rate of 15% and a complication rate of 12% (Cayan et al. 2009).

Meta-analysis data has shown that varicocoele repair in infertile men with nonobstructive azoospermia can improve semen analysis and spontaneous pregnancy rates (Weedin et al. 2010). Varicocelectomy for orchalgia secondary to a varicocoele has previously been studied, with studies demonstrating various success rates ranging from 48 to 88% (Biggers and Soderdahl 1981; Peterson et al. 1998; Kachrilas et al. 2014). We aim to evaluate the role of varicocele embolisation in the treatment of varicocele related scrotal pain, as this information is lacking in the literature.

## Methods

A multi-institutional, prospective study was performed over a period of 10 years. Patients over 16 years of age with a clinical diagnosis for varicoceles confirmed on ultrasound scan, who underwent varicocele embolisation for pain, were invited to participate in the study. Embolisations for male infertility were excluded. None of the patients had responded to prior conservative management. Patients who had other causes of scrotal pain, such as testicular torsion, epididymitis, orchitis, inguinal hernia or trauma were excluded. Local institutional approval was taken.

Patients were sent questionnaires evaluating pain scores, analgesia requirements and satisfaction scores of the procedure. Pain scores were evaluated with a 10 point visual analogue score (VAS), 0 = no pain and 10 = worse possible pain. Patients were asked to describe their pain when it was at its least, its most typical and when it was at its worst. Based on the most typical response patients were stratified into four main groups, no pain (VAS = 0), mild symptoms (VAS = 1–3), moderate symptoms (VAS = 4–7) and severe symptoms (VAS = 8–10). Pre and post procedure data were compared. Analgesia requirements were assessed, with patients divided into four main groups (never, rarely, often, always). Post embolisation questionnaires were collected within 12 months of treatment.

## Results

A total of 96 cases were identified. The mean age was 34 (17–75). 89% were located on the left side, 6% on the right side and 5% were bilateral. The mean number of coils used was 4 (2–8). The median time from embolisation to post operative questionnaire response was 12 months (95% CI).

**Fig. 1:**
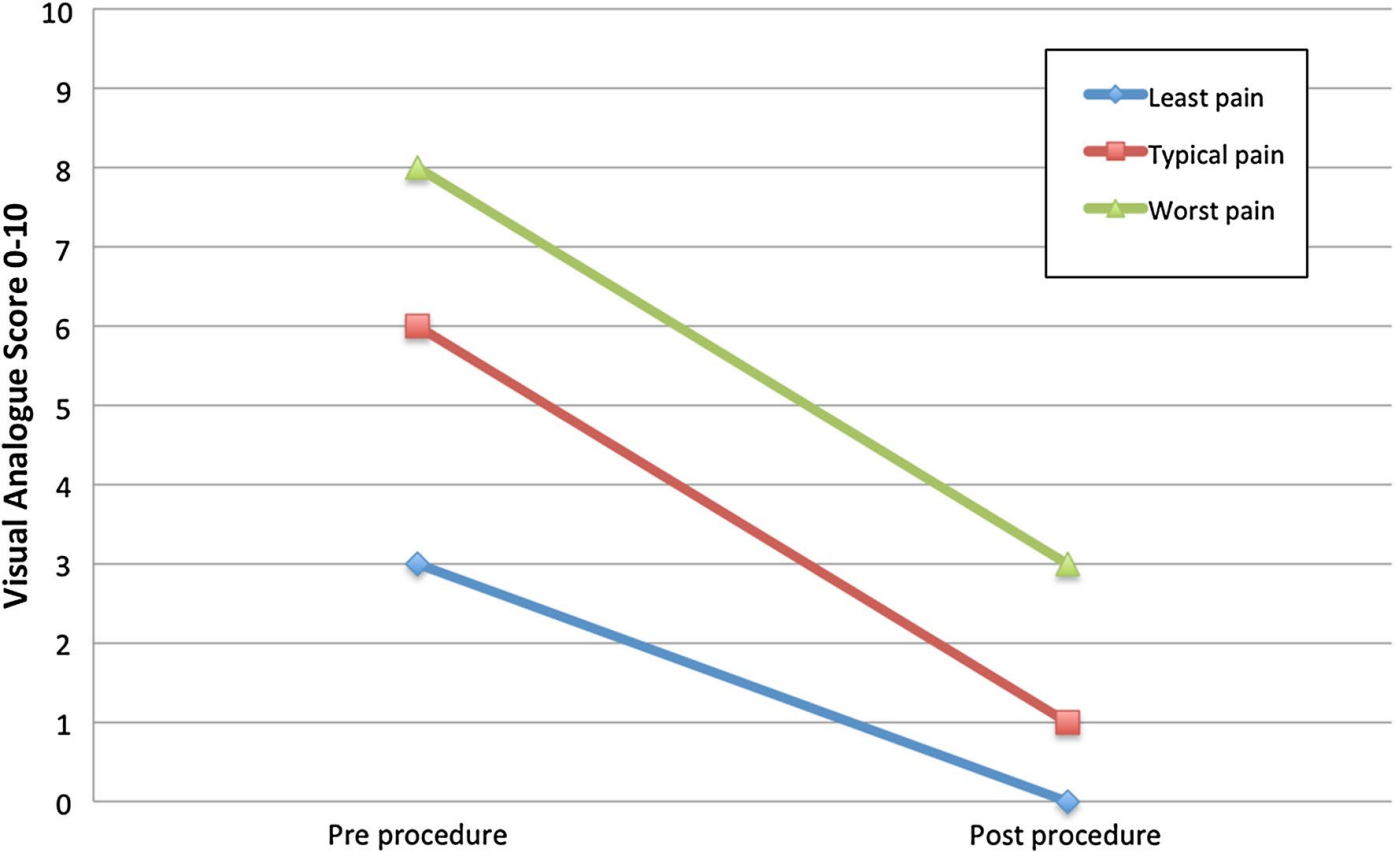
Pre and post procedure median pain scores.

Post-embolisation median pain scores reduced significantly in all three pain cohorts (least pain, typical pain and worst pain) when compared to the pre-embolisation pain scores (p < 0.001) (Fig. 1). Overall 74% of patients had improved pain post procedure, of which 30% had no pain following embolisation. 24% patients had no change in symptoms and 1% patient had worsening symptoms (Table 1).

**Table 1:** Varicocele embolisation outcomes compared to pre-embolisation symptom severity

Symptom severity	Resolution of pain (%)	Symptoms improved (but not resolved) (%)	No change (%)	Symptoms worse (%)
Mild	36	N/A	54	N/A
Moderate	38	43	19	0
Severe	15	64	21	N/A
Overall	30	44	24	1

The average post-procedure decrease in pain score in those with mild, moderate and severe pain was 1.2, 4 and 4.4 respectively.

**Fig. 2:**
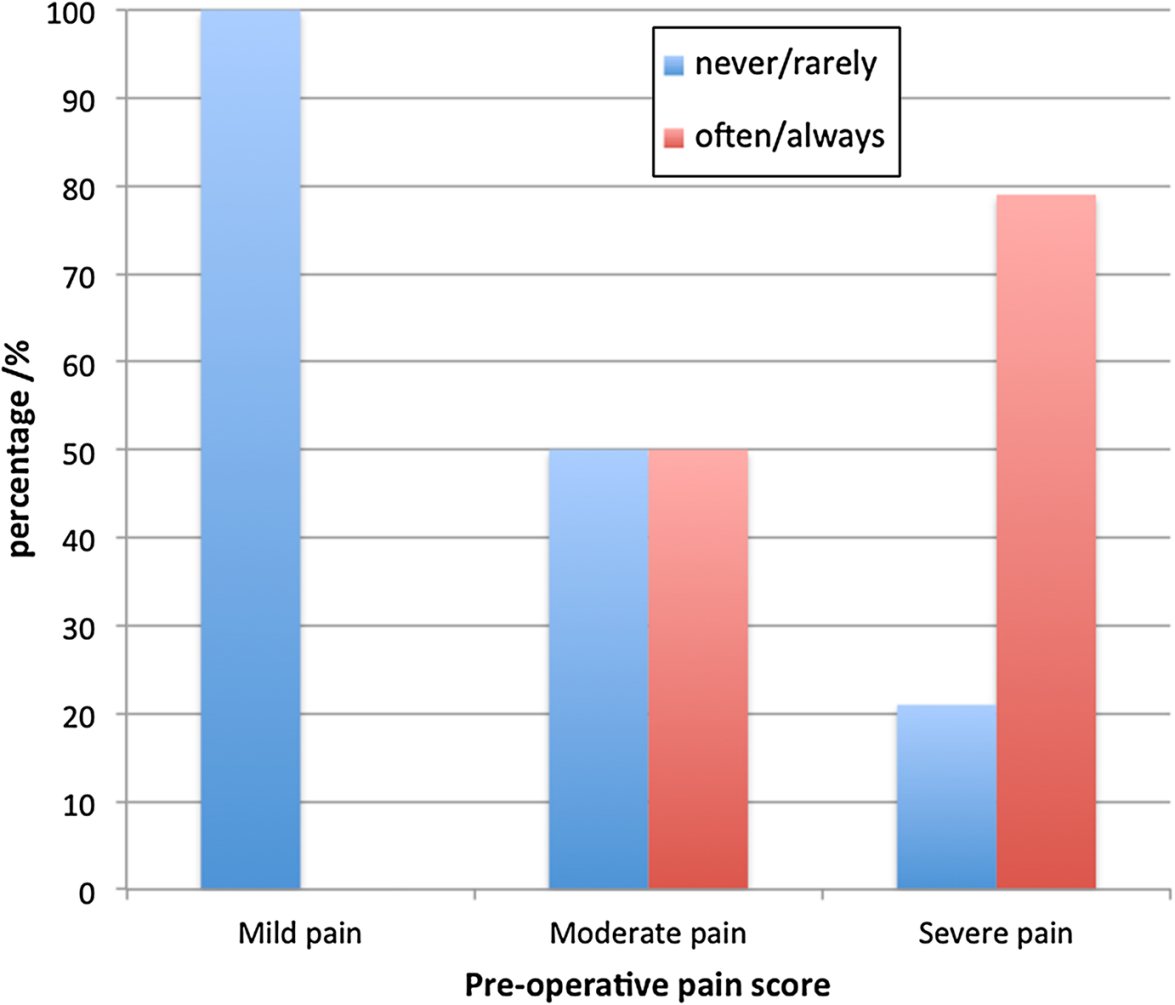
Pre-procedure analgesia requirements.

**Fig. 3:**
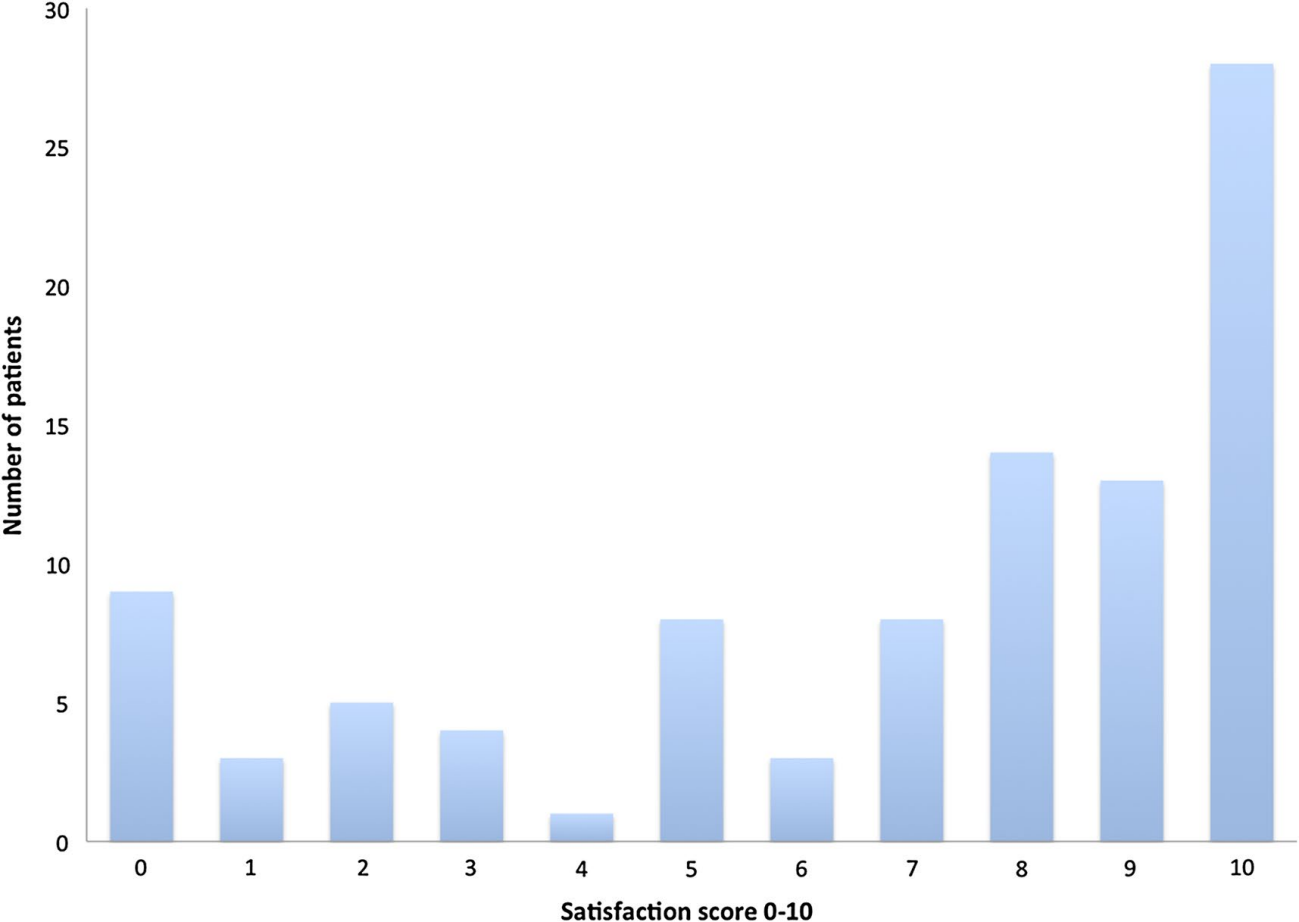
Post operative satisfaction scores.

Post-embolisation analgesia requirements were reduced when compared to pre-embolisation analgesia requirements. Those patients who often/always took analgesia for pain pre-embolisation experienced either moderate or severe pain (Fig. 2). Varicocele embolisation reduced this number from 51 to 20 (a reduction of 62%). Overall 53% of cases went from requiring some form of analgesia pre-embolisation to never requiring analgesia whatsoever. The overall median satisfaction score was 8 (VAS: 0–10, 10 = completely satisfied) (Fig. 3).

## Discussion

Multiple studies have examined the role of varicoceles treatment for subfertility. However the primary treatment of painful varicoceles through embolisation has not been widely investigated. From our literature review this is the first study to report the relationship between varicocele embolisation and orchalgia. With varicoceles being relatively common and a significant number of these patients experiencing pain, we need to be able to advise our patients on the best chance of success with treatment.

Standard management of patients with painful varicoceles would be a conservative approach, encompassing scrotal support and analgesia. If this fails then invasive treatments such as radiological embolisation or surgical ligation (open, microsurgical or laparoscopic) are considered. To date the role of embolisation for varicocele related orchalgia has been unclear, particularly whether technically successful embolisation of the varicocele actually improves pain.

We have demonstrated that varicocele embolisation can decrease scrotal pain in the presence of varicoceles with an overall reduction of pain in 74% of cases of which 30% were cured. This is relatively comparable with laparoscopic ligation (Kachrilas et al. 2014).

Our data suggests that embolisation success rates for pain seems to work best in those with moderate or severe pain, with an improvement of pain in 81 and 79% of cases respectively as well as a complete resolution of pain in 38 and 15% of cases respectively. 64% of patients who present with mild pain receive no benefit from embolisation and hence should be counseled appropriately.

One of the limitations of this study was the fact that we did not investigate the grade of varicocele and its relation to pain and treatment. One reason is because the clinical grading of varicoceles is based on physical examination, which can be quite subjective and can miss small sub-clinical varicoceles. It has been shown that colour Doppler ultrasound (CDUS) can accurately and reliably diagnose varicoceles and this is now regarded as the most robust investigation for diagnosing and grading varicoceles (Liguori et al. 2004). The grade of varicocele can possibly be an important prognostic factor for the success rate of treatment. It is already acknowledged that when choosing patients for treatment it is not recommended to consider patients with small or subclinical varicoceles for treatment, as this does not improve either pain or fertility rates (Iaccarino and Venetucci 2012). Also it is unclear whether the patients with severe symptoms had a proportionately higher grade of varicocele, and as such, successful treatment of these varicoceles could be more challenging. This could be a confounding issue that may affect our results. Furthermore one theory is that the true aeiology of varicocele related orchalgia is secondary to intratesticular varices. These are likely to be more symptomatic and more frequently found in higher grade varicoceles and as a result the likelihood of a better outcome in these cases is greater.

Another consideration for future studies would be an analysis of the duration of pain prior to embolisation. We were limited on this study, but the duration of pain could also be an important independent prognostic factor. A large retrospective study has looked at patients who had subinguinal varicocelectomy for pain. They found that if the pain had been present for >3 months then treatment had a higher success rate (98%), compared to patients who had short-term pain of <3 months (82.3%; p < 0.05)(Altunoluk et al. 2010).

In our study, varicocele embolisation was a well tolerated procedure with a median satisfaction score of 8/10. We were concerned about the biased caused by symptom severity and satisfactions scores. It could be argued that a small improvement in those cases with severe pain, leads to a subjectively greater response than those cases with mild pain. As a result the satisfaction seen with those patients would have been greater. However this was not found to be the case. Both groups with severe and mild symptoms pre-procedure had a median satisfaction score of 7 showing that the severity of the symptoms did not skew the satisfaction score.

In this study the mean number of coils used was 4 (2–8), which is comparable to the documented literature of 5 (Iaccarino and Venetucci 2012). However the variability of techniques was not established with certainty. Although there is national and international consensus on how this procedure should be performed, this study did not look into details about each operator’s technique. In addition the number of coils used could also be an independent prognostic factor. From this study it is unclear whether the number of coils used is related to the success rate of treatment and in turn improvement of pain. Further investigation into this area should be considered.

## Conclusion

In our review we have demonstrated that primary varicocele embolisation can decrease discomfort in those with symptomatic varicoceles, with an overall reduction of pain in the majority of cases. Patients with mild pain may not experience as much benefit as those with more severe pain. As such the severity of scrotal pain in the presence of varicoceles has been found to be an important independent prognostic indicator. Therefore the classification of patients into those with mild, moderate or severe symptoms prior to embolisation should be performed so robust consenting can be achieved.

## References

[CR1] Al-Said S, Al-Naimi A, Al-Ansari A (2008). Varicocelectomy for male infertility: a comparative study of open, laparoscopic and microsurgical approaches. J Urol.

[CR2] Altunoluk B, Soylemez H, Efe E, Malkoc O (2010). Duration of preoperative scrotal pain may predict the success of microsurgical varicocelectomy. Int Braz J Urol.

[CR3] Bechara CF, Weakley SM, Kougias P, Athamneh H, Duffy P, Khera M (2009). Percutaneous treatment of varicocele with microcoil embolization: comparison of treatment outcome with laparoscopic varicocelectomy. Vascular.

[CR4] Biggers RD, Soderdahl DW (1981). The painful varicocele. Mil Med.

[CR5] Cayan S, Shavakhabov S, Kadioğlu A (2009). Treatment of palpable varicocele in infertile men: a meta-analysis to define the best technique. J Androl.

[CR6] Iaccarino V, Venetucci P (2012). Interventional radiology of male varicocele: current status. Cardiovasc Intervent Radiol.

[CR7] Kachrilas S, Popov E, Bourdoumis A, Akhter W, El Howairis M, Aghaways I (2014). Laparoscopic varicocelectomy in the management of chronic scrotal pain. JSLS J Soc Laparoendosc Surg.

[CR8] Kass EJ, Marcol B (1992). Results of varicocele surgery in adolescents: a comparison of techniques. J Urol.

[CR9] Köse MG, Önem K, Çetinkaya M, L EK, Arpali E (2013). Influence of preoperative pain duration on microsurgical varicocelectomy outcomes. Adv Urol.

[CR10] Liguori G, Trombetta C, Garaffa G, Bucci S, Gattuccio I, Salamè L (2004). Color Doppler ultrasound investigation of varicocele. World J Urol.

[CR11] McAninch JW, Lue TF (2012) Smith & Tanagho’s General Urology, 18th edn

[CR12] Nabi G, Asterlings S, Greene DR, Marsh RL (2004). Percutaneous embolization of varicoceles: outcomes and correlation of semen improvement with pregnancy. Urology.

[CR13] Peterson AC, Lance RS, Ruiz HE (1998). Outcomes of varicocele ligation done for pain. J Urol.

[CR14] Weedin JW, Khera M, Lipshultz LI (2010). Varicocele Repair in patients with nonobstructive azoospermia: a meta-analysis. J Urol.

[CR15] Practice Committee of the American Society for Reproductive Medicine (2004). Report on varicocele and infertility. Fertil Steril.

