# Erratum to: The effectiveness of varicocele embolisation for the treatment of varicocele related orchalgia

**DOI:** 10.1186/s40064-015-1271-5

**Published:** 2015-09-07

**Authors:** David W Muthuveloe, Vinnie During, Daniel Ashdown, Nicholas J Rukin, Rob G Jones, Prashant Patel

**Affiliations:** School of cancer sciences, University of Birmingham, Birmingham, UK; Cambridge Health at Work, Cambridge Biomedical Campus, Hills Road, Cambridge, UK; The Royal Wolverhampton Hospital NHS Trust, Wolverhampton, UK; Interventional Radiology Department, University Hospitals Birmingham, Birmingham, UK

## Erratum to: SpringerPlus (2015) 4:392 DOI 10.1186/s40064-015-1177-2

It has come to our attention that during production of the original article, an error was introduced into Table 1 during copyediting. The corrected Table 1 can be found below. The publisher apologises for inconvenience caused.

**Table 1:** Varicocele embolisation outcomes compared to pre-embolisation symptom severity

Symptom severity	Resolution of pain (%)	Symptoms improved (but not resolved) (%)	No change (%)	Symptoms worse (%)
Mild	36	N/A	54	10
Moderate	38	43	19	0
Severe	15	64	21	N/A
Overall	30	44	24	1

